# Public backlash against science communicators: Conceptualization and qualitative analysis of perceptions, effects, responses, and contextual factors

**DOI:** 10.1177/09636625261452891

**Published:** 2026-06-03

**Authors:** Niels G. Mede, Sophia C. Volk

**Affiliations:** 1 Strategic Communication Group, Department of Social Sciences, Wageningen University & Research, The Netherlands; 2 Department of Media and Communication, Ludwig-Maximilians-Universität München, Germany

**Keywords:** science communication, public engagement with science, harassment, hate speech, qualitative interviews, university communicators

## Abstract

Public backlash against scientists is drawing growing concern. It can come from citizens, politicians, or journalists and ranges from legitimate criticism to harassment and physical attacks. Backlash also affects science communication professionals, yet this has received comparatively little public and scholarly attention. Our study addresses this gap. Drawing on qualitative interviews with 15 university communicators from multiple countries, we develop a conceptual model of their perceptions of public backlash, its effects on them and their institutions, their response strategies, and contextual factors. We introduce criteria for distinguishing legitimate criticism from illegitimate harassment and describe both negative and positive effects on science communication. The results show proactive and reactive response strategies and highlight the influence of topic characteristics, routes of backlash, and sociopolitical contexts. Overall, they offer a new perspective on the normative imperative for public engagement. We discuss implications for science communication research and practice.

## 1. Introduction

Public outreach and engagement by science communicators are essential in knowledge societies: they feed scientific expertise into individual and collective decision-making, facilitate dialogue and participation, and help maintain the public legitimacy of science (Bucchi and Trench, 2021). Communication professionals at universities—the focus of this study—have taken on a particularly important role at the science–society interface, as universities have substantially intensified their communication efforts (Fürst et al., 2022a).

Science communication often elicits affirmative public feedback (Volk et al., 2025). However, it can also provoke backlash—particularly on politicized issues such as climate change or vaccination, as exemplified by the Climategate controversy (Ramírez-i-Ollé, 2015) and hostility toward epidemiologists during the COVID-19 pandemic (Nogrady, 2021). Such backlash comes from citizens, politicians, journalists, or other scholars (Peetz et al., 2024) and occurs in online, journalistic, or face-to-face communication (Seeger et al., 2024). It ranges from criticism of errors or misconduct (Prasad and Ioannidis, 2022) to intimidation, insults, harassment, threats, and physical attacks (O’Grady, 2022). Backlash at the severe end of this spectrum poses a serious challenge to scholars’ well-being, careers, and professional and social environments, and to the societal status of science (Nölleke et al., 2023).

Public backlash targets not only scientists, but also science communication professionals. Harvard University’s communication office, for instance, faced substantial pushback over its handling of plagiarism and antisemitism accusations against president Claudine Gay in 2023 and 2024 (Hall, 2024). Even when not the direct target, communication practitioners at higher education and research institutions are often “in the line of fire” (O’Grady, 2022: 1338), mediating between attackers and targets as “boundary spanners” (Schwetje et al., 2020: 193). University communication offices, for example, frequently support scientists under attack by offering preventive training and helping them cope with aftermath (Peetz et al., 2024). Communicators themselves may thus suffer negative consequences through both direct and indirect experiences of criticism and harassment.

However, the double burden on science communication professionals—as both targets and bystanders of public anti-science backlash—has been underacknowledged and understudied (Soltis, 2024). Neither conceptual models nor systematic empirical evidence exist for professionals’ (1) *perceptions* of backlash, (2) attitudinal, individual, organizational, and systemic *effects*, (3) proactive and reactive *responses*, and (4) *contextual factors* shaping perceptions, effects, and responses. This limits science communication research’s ability to provide practitioners with evidence-informed recommendations for effective countermeasures (Nogrady, 2024). We address this gap through a qualitative interview study with 15 experienced university communicators in nine countries.

Our study is among the first to examine perceptions, effects, responses, and contextual factors of public criticism and harassment in science communication practice. Unlike most existing research, our conceptual approach and design integrate both illegitimate and legitimate criticism and consider both negative and positive effects. We also develop a conceptual model that systematizes the dimensions of public backlash, examines their relationships, and helps organize future research and practice.

## 2. Literature review

### Public backlash against science communicators

The public sphere, a “realm of our social life in which something approaching public opinion can be formed” (Habermas et al., 1974: 49), invites various actors in society to discuss and criticize science. These include scientists, citizens, journalists, politicians, and communication practitioners working at the science–society interface, for example at higher education and research institutions (Galpin and Vernon, 2024).

Conceptions of science communication in the public sphere rest on different normative views of what constitutes legitimate or illegitimate public criticism (see Eisenegger and Schäfer, 2023). *Deliberative* conceptions emphasize factual argumentation, civil tone, and equal participation (Dahlberg, 2001), whereas *agonistic* conceptions hold that incivility and aggression may also be valuable qualities of public discourse about science (Spoel et al., 2008; see also Mede and Schäfer, 2020). Social movement research suggests a similar distinction between *normative* and *non-normative* forms of public engagement with science (see Zlobina and Gonzalez Vazquez, 2018). Normative engagement uses peaceful, legal, and “morally acceptable means of criticizing” the status quo, whereas non-normative engagement involves illegal, violent, and thus illegitimate actions (Teixeira et al., 2023: 2). Binary conceptions of deliberative versus agonistic public spheres and normative versus non-normative public engagement can be criticized (see Uysal et al., 2024), but they remain useful for our study because they map the spectrum of legitimate versus illegitimate backlash against epistemic authorities, including scientists and science communication professionals.

The term “backlash” is used sporadically and inconsistently in the science communication literature, typically without theoretical explication (e.g. Verovšek and Gorišek, 2023: 496). We use it here as an umbrella term for the various speech acts and behaviors aimed at challenging, contesting, disparaging, or harming three analytically distinct targets: individual scientists and science communication professionals, science institutions such as universities, and science as an epistemic system.^1^ Backlash therefore encompasses constructive criticism as well as destructive harassment, including hybrid forms in which the two intersect, such as criticism of vested interests in industry-funded research voiced through profanity. While no single term may fully capture the spectrum of negative public reactions to science, “backlash” is arguably the best available shorthand of the range of phenomena we analyze in this study.

Research has examined several forms of public backlash against science across disciplines and world regions (Economist Impact, 2022; Gerards and Schattevoet, 2024; Global Witness, 2023). *Normative* backlash that aligns with *deliberative* ideals includes criticism of unintended errors, vested interests with funders, self-profit seeking, and scientific misconduct, for example (Hesselmann and Reinhart, 2019; Johnson and Dieckmann, 2020; Mede et al., 2025). Attacks on scientists’ credibility and allegations of dishonesty may cross the line to *agonistic* science communication, especially when they involve profanity and insults (Anderson and Huntington, 2017; Peters, 2024; see also Kim et al., 2022). Further research documents racist and sexist assaults, death threats, physical attacks, and vandalism, that is, *non-normative* forms of backlash (McDonald et al., 2020; Medina et al., 2021; Väliverronen and Saikkonen, 2021a). These are often at odds even with agonistic approaches to science communication, which nonetheless subscribe to democratic values and reject illegal behavior (Mouffe, 2002).

Agonistic and non-normative forms of backlash pose a particular challenge to science communication (Väliverronen and Saikkonen, 2021b). They may compromise the well-being, willingness to speak publicly, reputation, and trustworthiness of science communicators, for example (Celuch et al., 2024; Nölleke et al., 2023; Oksanen et al., 2022). Our study therefore centers on these forms, which we summarize as “harassment,” an analytical category that has been used in prior ad hoc research (O’Grady, 2022), theorized in related fields such as journalism (Miller, 2023) and political communication research (Kümpel and Unkel, 2023), and applied within science communication research itself (Egelhofer et al., 2024).

### Conceptualizing harassment in science communication

Originating from scholarship on “negative verbal or non-verbal behavior” at the workplace (Saunders et al., 2007: 342), the term “harassment” has been applied across disciplines, including science communication research. However, it is often used as an umbrella category for a variety of verbal attacks, varying in severity and ranging from rude credibility attacks to insults targeting scientists’ personal characteristics, such as their physical appearance, and in more extreme cases to threats of physical or sexual violence and even death threats (Egelhofer et al., 2024: 2).

It is therefore helpful to draw on adjacent fields such as journalism research, where there is “a relative consensus to the use of ‘harassment’” (Miller, 2023: 1232) to describe “unwanted, abusive behaviors of mild to extreme kinds of verbal or physical abuse” (Menke and Seeger, 2026: 85). This approach sidesteps uncertainty about source intentions, focuses on target perceptions, and avoids normative categories such as “aggressive” or “worthwhile,” thereby accommodating both normative and non-normative behaviors.

Political communication research further distinguishes the arguments and tone of harassment, such that harassers may voice legitimate arguments while using uncivil tone (Kümpel and Unkel, 2023). This distinction helps disentangle differential normative perceptions of public backlash against science. Scholarship on conceptions of toleration adds further nuance: the “permission conception” suggests that targets endure backlash even when they disagree with it, while the “esteem conception” suggests that targets may value backlash despite disagreeing (Forst, 2017). Unlike harassment of politicians, however, harassment of science communicators centers on truth claims and the epistemological and ethical norms of science rather than on political authority and power (see Mede et al., 2025). Unlike journalists, science communicators are often not direct targets but bystanders (Stahel and Schoen, 2020). And unlike influencers or opinion leaders on social media, they are typically affiliated with institutions and professional associations, which adds another layer of specificity to harassment in science communication contexts (McDonald et al., 2020).

Beyond this conceptual blurriness, existing research has three further shortcomings. First, it has focused on communicating scientists and largely overlooked communication professionals at higher education and research institutions (e.g. Gosse et al., 2021; Grimes et al., 2020; Nölleke et al., 2023; Väliverronen and Saikkonen, 2021a). One exception is McDonald et al. (2020), who examined gender-based harassment of science bloggers and online influencers, but did not consider institutional settings, which have become increasingly important in science communication (Fürst et al., 2022a). Second, evidence on perceptions, effects, and responses of communication professionals facing backlash is scarce, as existing studies rely on media analyses rather than self-report data (Chen et al., 2023; Peters, 2024; Soltis, 2024). Third, much of the literature does not explore the tipping points between legitimate and illegitimate forms of backlash, even if legitimate backlash also warrants scrutiny, as we otherwise risk pathologizing potentially valuable forms of critical opinion and communication about science (Krause, 2023). Our study addresses these gaps by (a) focusing on communication professionals at universities, (b) using qualitative interviews to provide nuanced evidence on perceptions, effects, and responses, and (c) examining not only non-normative and agonistic, but also normative and deliberative forms of public backlash.

### Types, effects, responses, and contextual factors of anti-science backlash

Research has identified different dimensions of backlash against scientists and science communicators. We follow the common approach to distinguish the four dimensions *types*, *effects*, *responses*, and *contextual factors* (see Gosse et al., 2021).

#### Types of backlash

Grimes et al. (2020) propose a taxonomy of different types of “common adversarial tactics” against medical science communicators. It describes several forms of non-normative backlash, including insults, threats, discreditation attempts, and physical attacks (Nogrady, 2021; O’Grady, 2022; Peetz et al., 2024). The taxonomy also includes potentially legitimate criticism of scientists—for example, skepticism about vested interests and scrutiny of professional capabilities—and is thus one of the few attempts to integrate normative forms of public backlash. However, Grimes et al. (2020) do not consider many other normative forms, such as public concerns about ethical misconduct, perceived undue activism, or scientific elitism (Cologna et al., 2025; Hesselmann and Reinhart, 2019; Scheufele, 2022). They also offer little insight into how science communication professionals perceive backlash directed at their institution or its members, which presumably has different qualities than backlash against themselves. Other studies that have analyzed backlash perceptions typically measure perceived quantity rather than perceived intensity (e.g. Science Media Center Spain, 2024). An exception is Verhaeghe (2023), whose survey on public harassment and intimidation of Flemish scientists used a mild-to-severe spectrum of perceived severity. Our first research question explores such perceptions:

**RQ1:** How do science communication professionals perceive different types of public backlash against them, their institutions, or colleagues at their institutions?

#### Effects of backlash

Several studies have analyzed the effects of public criticism and harassment on science communicators. Cognitive effects include self-blame and distraction from work routines, as well as increased knowledge about how to cope with harassment (Celuch et al., 2022; Veletsianos et al., 2018). Affective effects include frustration, stress, anxiety, fear of reputational loss, and mental illness, but also increased resilience and agency (Gosse et al., 2021; Nölleke et al., 2023). Behavioral effects involve adapting outreach strategies and self-censorship, as well as reinforced motivation to engage in science communication (Celuch et al., 2024; Chen et al., 2025; Marcinkowski et al., 2025; Väliverronen and Saikkonen, 2021a). Most studies, however, have focused on scholars and have not accounted for effects on organizations, such as reputational damage (Fürst et al., 2022b). We explore perceived backlash effects from the perspective of professional science communicators:

**RQ2:** What are the perceived effects of public backlash against science communication professionals, their institutions, or colleagues at their institutions?

#### Responses to backlash

Our third research question asks how targets and bystanders of public criticism and harassment respond to it. Conceptually, our analysis draws on Veletsianos et al.’s (2018) distinction between reactive, anticipatory, preventive, and proactive coping strategies among female scholars experiencing online harassment. We focus on reactive and proactive strategies, which are the primary focus of current institutionalized responses to attacks on science (Researcher Support Consortium, 2024; Scicomm Support, 2023; WetenschapVeilig, 2023). Previous studies have mapped various coping strategies, such as avoiding research topics and digital platforms where harassment is more likely to occur (Seeger et al., 2024). Other responses include reporting or blocking harassers on social media, seeking emotional support from family or colleagues, consulting psychologists, and taking legal action (Houlden et al., 2022; McDonald et al., 2020; O’Grady, 2022). Some studies describe less proactive behaviors such as ignoring or accepting backlash (Veletsianos et al., 2018). We address a major caveat of these studies, which is that few of them provide evidence on institutional responses, such as crisis communication and reputation management in university communication offices (Taylor et al., 2025).

**RQ3:** How do science communication professionals respond to public backlash against them, their institutions, or colleagues at their institutions?

#### Contextual factors

The types, effects, and responses to backlash against science communicators are shaped by a range of contextual factors. Scholars with high public visibility who study politicized topics—such as climate change, COVID-19, or gender issues—are disproportionately prone to harassment (Global Witness, 2023; McDonald et al., 2020; Nogrady, 2021; Rodriguez et al., 2025). Perceptions, effects, and responses also differ depending on where backlash occurs: social media, particularly platforms with high degrees of anonymity and pseudonymity, have been identified as arenas of non-normative backlash (Seeger et al., 2024). Repercussions are often more severe when harassment is networked or coordinated (Doerfler et al., 2021) and when it occurs at in-person events (Väliverronen and Saikkonen, 2021a). Moreover, perceptions, effects, and responses may differ depending on individual characteristics and dispositions, such as gender, age, race, career stage, or emotional resilience (Celuch et al., 2022; Gosse et al., 2021; Peetz et al., 2024).

Little research has explored institutional-level factors, such as the availability of support structures, which can affect how targets anticipate or react to backlash (Researcher Support Consortium, 2024). Society-level factors, such as normative conceptions of science communication, can also shape perceptions, effects, and responses (Fischer et al., 2025), but are similarly underresearched. Our fourth research question addresses this gap:

**RQ4:** Which individual, organizational, and society-level factors contextualize perceptions, effects, and responses to public backlash?

## 3. Data and method

### Participants

We conducted semi-structured in-depth interviews with 15 experienced university communicators in 9 countries across 4 continents. Participants were recruited through a two-step convenience sampling approach (Parker et al., 2020). First, we drew on a quantitative survey conducted in late 2022 among members of the European Association of Communication Professionals in Higher Education (EUPRIO) for another project (*N* = 206). One section of the survey asked respondents about their experience with public backlash and their interest in participating in a follow-up study. The results indicated that university communicators had encountered backlash of various kinds: 11% had very often experienced attacks on the credibility of their university or its members, including themselves, and 5% had very often experienced emotional or physical aggression. Frequent threats of violence or actual violence were rarely reported. In 19% of cases, the university as a whole was frequently targeted, followed by scientists (16%); only 1% reported that the communicators themselves were very often targeted.

Second, we distributed a 3-minute screening survey containing the backlash-related items from the EUPRIO survey through professional associations of university communicators (e.g. the Public Communication of Science and Technology Network), newsletter lists (e.g. The SciCommer), and social media groups (e.g. the African science communication group on Facebook). In total, 35 respondents signed up for an interview. We excluded those who did not report direct or indirect backlash experiences and those who did not work as communication professionals at or with a university or research institute.

The final sample consisted of 15 communicators from Australia, Austria, Canada, Germany, Kenya, the Netherlands, Sweden, Switzerland, and the United Kingdom (see Supplemental Material, Appendix 1); 10 identified as female. They worked at public universities, a research institute, or independently for multiple universities and scientists. One worked as a science communication practitioner and coordinator for non-profit initiatives. Most institutions had at least occasional experience with public backlash, much of it gained during the COVID-19 pandemic, and dealt with it as part of their broader crisis communication work. However, few had institutionalized, formalized procedures or dedicated staff for handling it. All participants gave written informed consent before the interview. They received no remuneration but retained early-access rights to study results.

The sample of 15 is small in absolute terms but appropriately scaled to a specialized population—university communication professionals with direct or indirect backlash experience—which is itself comparatively small, and we reached sufficient saturation across our analytical categories. The international scope allowed us to examine nine countries with varying levels of anti-science backlash, forms of political interference with academia, and normative approaches to science communication (Economist Impact, 2022; Fischer et al., 2025; Gerards and Schattevoet, 2024; Global Witness 2023), and to account for the context-specificity of backlash and countermeasures across science communication cultures. For example, university communication in the United Kingdom and the Netherlands may be better prepared against backlash, as it tends to be strongly institutionalized, but also more backlash-inviting, as it tends to be more public-serving (Fischer et al., 2025). At the same time, the study design reduces the depth of insight we can offer for any single national setting.

### Data collection and instrument

Data collection took place between March and September 2023. The authors conducted all interviews via online video calls, except for five conducted by extensively trained undergraduate students. The interviews followed a collaboratively developed semi-structured guide covering all aspects of our research questions (see Supplemental Material, Appendix 2). We asked participants about their workplace and function, professional role conception, backlash perceptions (i.e. types, sources, targets), routes through which they received backlash (e.g. media, events, topics), perceived consequences, coping strategies, and support measures.

**Figure 1.**
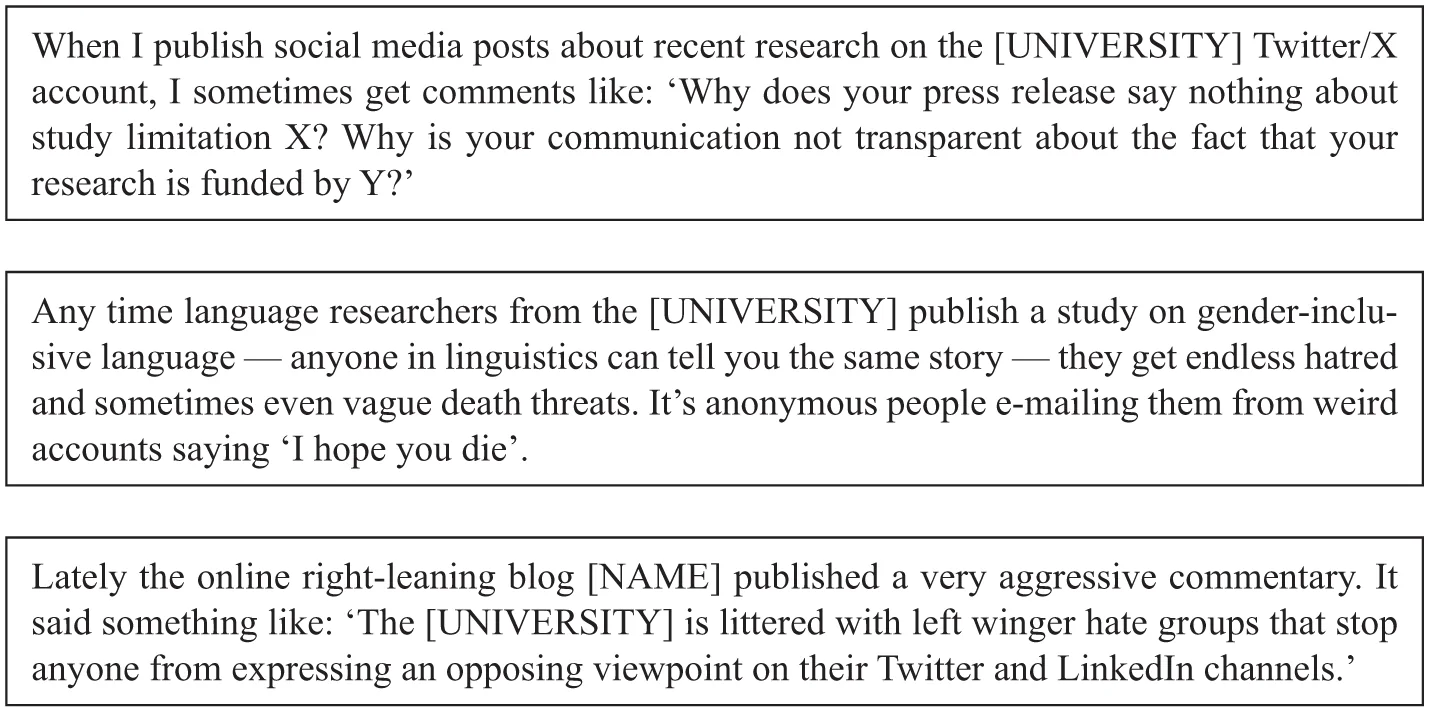
Vignette showing different forms of criticism and harassment.

The interview included a vignette prompting respondents to reflect on three hypothetical examples of backlash, assess their severity and legitimacy, and indicate whether they had experienced or observed such incidents (see Figure 1). The examples illustrated potentially legitimate criticism of study limitations, skepticism about political bias, and death threats, spanning the spectrum from normative and deliberative to non-normative and agonistic forms of backlash. They were adapted to each interviewee’s context. Participants were informed that the examples were fictional but based on real cases. The interview guide and vignette were piloted and refined in one test interview with a science communicator.

Interviews lasted on average 60 minutes and were audio-recorded for analysis. Transcripts were generated with an AI-based transcription tool and carefully checked by a trained research assistant. Interviewees were offered the opportunity to review their transcripts, most accepted, and a few made small corrections.

### Analysis

We applied structured qualitative content analysis to all interviews using MAXQDA (Rädiker and Kuckartz, 2019). The codebook was developed collaboratively, combining deductive and inductive coding: new categories were generated inductively and subsumed under broader, deductively derived main categories (see Supplemental Material, Appendix 3). To ensure reliability, the authors initially double-coded several transcripts independently. Discrepancies were jointly discussed and resolved, leading to refinements in the category system and coding rules. To enhance validity, the authors continuously compared the data and categories against the literature, ensuring that interpretations remained both informed by the theoretical background of the study and grounded in the empirical material (see Supplemental Material, Appendix 4 for interview excerpts including codings).

## 4. Results

**Figure 2.**
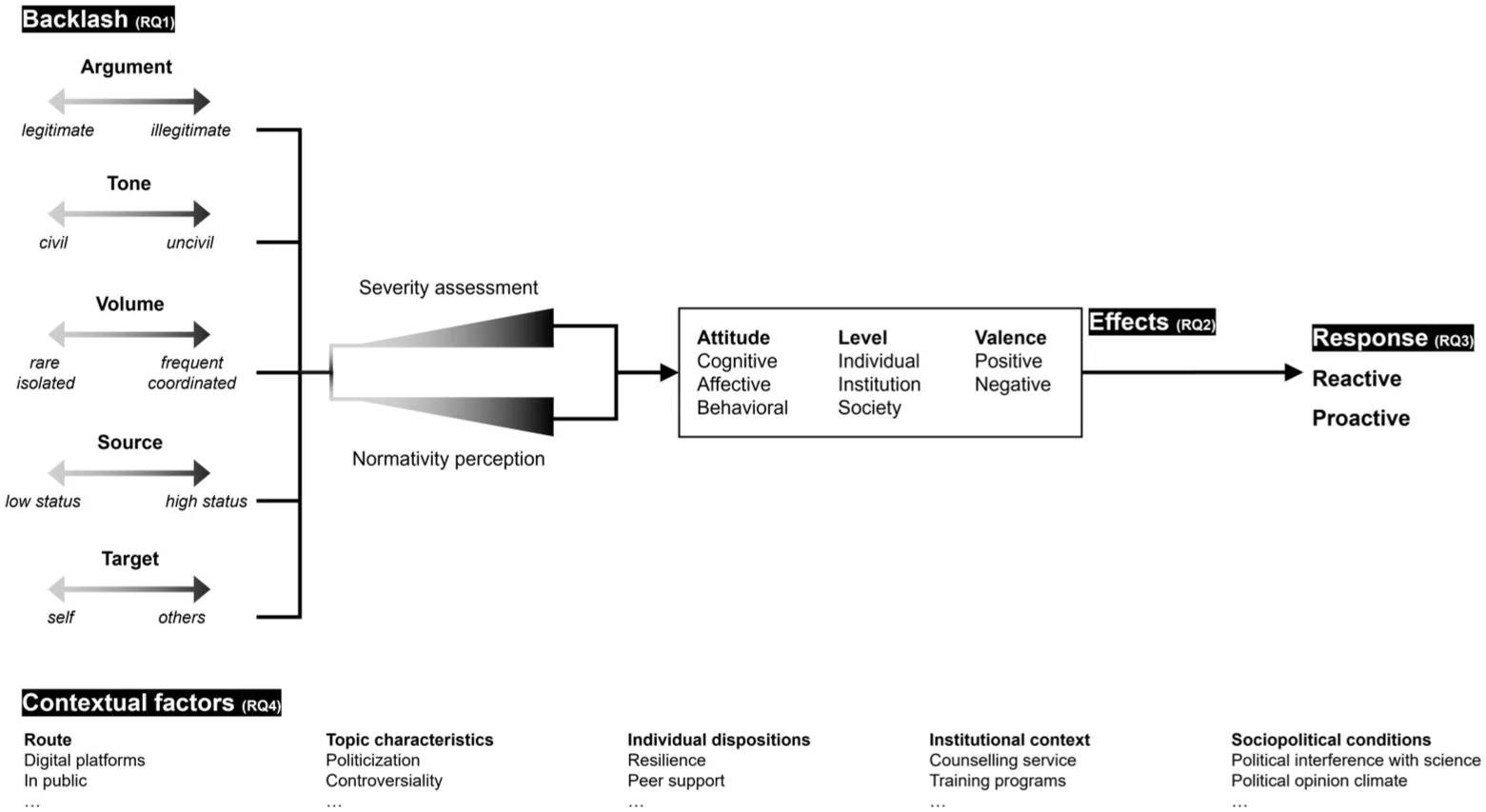
Model of perceptions, effects, responses, and contextual factors of public criticism and harassment in science communication.

Our analyses addressed three objectives. First, we explored perceptions (RQ1), effects (RQ2), and responses (RQ3) to public backlash against science communication professionals, their institutions, and other members of their institutions, and contextual factors (RQ4). Second, we identified common themes and patterns across participants, institutional settings, and countries. Third, we synthesized our observations into a conceptual model showing how the multiple dimensions of criticism and harassment produce a complex array of legitimacy assessments, impacts, and coping strategies (see Figure 2).

### Science communicators’ perceptions of backlash (RQ1)

The interview data suggest five dimensions of criticism and harassment: arguments, tone, volume, sources, and targets. We detail these dimensions and their interactions below.

#### Unbalanced dialectic of arguments and tone of public backlash

Participants reported several types of criticism and harassment, including allegations of incompetence, fraud, and ideological bias, as well as defamation, intimidation, hate, sexism, and death threats. One participant reported physical violence against themselves and their colleagues. Most of these forms have also been documented in the literature on harassment of scientists (e.g. Nogrady, 2021), journalists (e.g. Miller, 2023), and politicians (e.g. Krook and Restrepo Sanín, 2020). However, we also identify backlash characteristic of science communication, such as a rejection of the epistemic authority of science (Egelhofer et al., 2024), and of science communication at higher education and research institutions in particular, such as attacks on the reputation of universities.

Our analysis suggests that it is important to distinguish between the *arguments* and the *tone* of backlash. Arguments perceived as illegitimate typically rely on conspiracy narratives and ideological rationales. These clearly outweigh arguments perceived as legitimate, such as criticism of allegedly undue advocacy and activism by researchers, personnel recruitment strategies of university leaders, study limitations, and funding sources. Such criticism was often appreciated by participants; many exhibit “esteem toleration” (Forst, 2017). One participant noted: “The first vignette example would be absolutely legitimate criticism. Transparency of sources and limitations, that’s precisely what we should do” (Germany, I01). A few participants also perceived more severe accusations as legitimate, such as universities being part of a left-leaning, liberal elite. Prompted by the experimental vignette describing such accusations, one participant said: “In principle, this is criticism that deserves to be heard and must be scrutinized” (Germany, I01).

Backlash perceptions do not only involve assessments of argumentative substance, but also—and primarily—the *tone* of engagement. Participants described a wide variety of intolerable communication styles, ranging from “confusing, miserably long emails, not particularly offensive or dangerous, but clearly freaky” (Switzerland, I03) to aggression, insults, threats, and racial or sexist slurs (Sweden, I13). Some participants nevertheless emphasized that much public engagement, particularly with journalists, politicians, and other scholars, uses a civil tone and complies with politeness norms (see Kümpel and Unkel, 2023).

Overall, few participants elaborated on specific *arguments*. Most participants, including a communication professional at a major German university, focused on the *tone* of engagement:

It’s the tone: Obviously, threats to physical harm or death obviously go too far, and insults are not acceptable. Substantive critical discussion is always okay—debating is always okay. But as soon as it becomes insulting, abusive, or threatening, it is no longer okay. (Germany, I01)

This suggests that criticism and harassment in science communication typically revolve around perceived violations of politeness norms rather than violations of rational reasoning (see Egelhofer et al., 2024). Only a few participants explicitly distinguished between arguments and tone, indicating that the two have a dialectic relationship in which they are often too interrelated for participants to meaningfully separate. The argument–tone distinction—a prominent theme in the literature on hate speech—therefore appears to draw primarily from ex-post theorizing rather than empirical observation. We did, however, find exceptions: one participant identified “ideological reasons or reasons of self-identity” and “conspiracy theories” as the “crux of their critique,” explicitly distinguishing these from ad hominem attacks and gaslighting as stylistic devices used to articulate them (Canada, I07).

Overall, tone outweighs argumentation as a determinant of severity assessments and normativity perceptions; the argument–tone dialectic is therefore unbalanced (see Figure 2). For example, most participants perceived legitimate arguments delivered in an uncivil tone as non-normative backlash more often than they did illegitimate arguments delivered in a civil tone. This suggests that they may see incivility and profanity as a greater threat to science communication than conspiracy narratives and anti-scientific reasoning.

#### The volume of criticism and harassment matters

A third dimension of backlash perceptions is the *volume* of criticism and harassment. Almost all participants indicated that harassment is rare: “The cases that I handle in my organization are usually more a one-time thing that has a peak and then it slows down again.” (Netherlands, I11). Another participant quantified the volume of affirmative and consensual public feedback relative to negative backlash:

Our individual consulting is 99.9 percent about positive visibility [. . .]. Then we have that 0.1 percent or maybe 0.4 percent where we focus on what we’ve spent our time talking about in this interview. (Austria, I02)

Several participants described backlash as a function of publicity: criticism and harassment will inevitably occur once scientists and universities engage in outreach. According to one participant, publicity and backlash have a linear relationship:

If you are out there twice as much, there will be twice as many negative [reactions], but the percentage will be about the same. However, you have more impact overall because you are more present. [. . .] That’s the mechanism. (Switzerland, I03)

Contrary to common concerns, we found few mentions of coordinated or organized backlash (Marwick, 2021; see also Doerfler et al., 2021). One exception was a participant who described animal rights networks campaigning against research in a primate laboratory. Such backlash, even when using legitimate arguments and a civil tone, is often challenging, particularly when it occurs on a daily basis. Conversely, backlash using illegitimate arguments and an uncivil tone is typically perceived as less challenging when it occurs rarely. This illustrates that volume—including both the frequency and coordination of backlash—can be understood as another key dimension of perceived severity and normativity.

#### The relationship of source status and target vulnerability

Two further dimensions shape how communication professionals assess the severity and normativity of backlash against them or their institutions: the *source* and the *target*. We found that the large majority of backlash comes from anonymous sources—typically members of the general public, such as social media users—with no or weak ties to those they criticize or harass. Most participants consider backlash from anonymous sources less severe. Some, however, reported cases in which critics and harassers disclosed their identity at public events or had been working with the individuals and institutions they attacked:

It’s anonymous channels or anonymous profiles saying things. But it can also actually come from contacts that actually know the person from years ago or from previous relationships, either work or private relationships. (Netherlands, I11)

Backlash does not only come from an often undefined general public. Across all interviews, we found mentions of journalists, politicians, civil society groups, and other scientists interfering with participants’ outreach and communication efforts. For example, participants from the United Kingdom and Australia described unpleasant incidents with journalists, such as when a news reporter seemingly misinterpreted a word in a fact sheet and was then “threatening to publish a story that had my name on it” (Australia, I04).

Two observations stand out. First, backlash is typically perceived as more severe when it comes from sources with high status and reach, that is, actors with substantial political, financial, epistemic, or discursive power. Several participants noted that backlash from politicians and journalists may be particularly consequential because it often generates high public visibility. Second, criticism and harassment are especially challenging when they occur on multiple fronts at once:

It was really a major scandal. [. . .] We actually received threats. The hostility was against the whole university. The communications office was sort of in the middle: On one hand, coworkers were really upset about getting dragged into the scandal without being involved in this research. And on the other hand were the general public and the journalists, so we were in the middle. (Sweden, I13)

Participants’ perceptions of backlash severity also involve considerations about the target. Across all interviews, criticism and harassment were typically directed at institutions or individual scientists: “So far it has always been aimed at either the institution, like the university or the faculty or a research group, or individual researchers and colleagues. But luckily I haven’t experienced it personally” (Netherlands, I11). There were, however, isolated cases of attacks against science communication practitioners, especially when they represent the institution to the public. In these cases, practitioners may function as scapegoats:

We used to have a number of public forums so that the community there is sensitized and [. . .] they know the objective and the face of the science communicator. [. . .] When something goes wrong, the person that will be attacked, whether within the area or when somebody meets you in a different city or different town, is the person who came and was used as a guinea pig. The researcher is not there at the assembly. The person that will be targeted is the science communicator and the journalist that he was working with. [. . .] The person that will remain the face of that project is the science communicator and therefore, if anything goes bad, it is him or her. (Kenya, I12)

More often than individual communication practitioners, communication departments or teams—and in a few cases, other units of the university, such as the Diversity and Equality team—are targets of public backlash:

It was very, very intense when we published a declaration against racism [. . .]. We were also personally attacked as communicators. It came as an anonymous message to the team via Twitter. (Germany, I01)

### The differential effects of criticism and harassment (RQ2)

Our analyses on the effects of backlash against science communicators, their colleagues, and their institutions identify an attitudinal, a system-level, and a valence dimension. Each dimension depends, first, on participants’ assessments of backlash severity, that is, the degree to which criticism and harassment threaten or benefit their own well-being, that of their colleagues, their institutions, or society at large (see Figure 2). Second, the effect dimensions are shaped by normativity perceptions, that is, the degree to which criticism and harassment articulate legitimate or illegitimate arguments, use civil or uncivil tone, occur rarely or frequently, originate from low- or high-status sources, and target oneself or others.

#### Cognitive, affective, and behavioral effects (attitudinal dimension)

When asked about the consequences and impacts of public criticism and harassment, participants described a range of *attitudinal* effects on themselves, many of which are documented in the literature on harassment of scientists and journalists (Menke and Seeger, 2026; Nölleke et al., 2023). We distinguished cognitive, affective, and behavioral effects, building on established conceptualizations of how crisis situations affect the work of public relations practitioners (Jin and Cameron, 2007).

One notable cognitive effect is that public backlash has led some participants to question their efforts—and a broader imperative in science communication practice—to stimulate motivation, generate enthusiasm, and set reputational and monetary incentives for successful public engagement:

It’s a double-edged feeling because as a press officer and as a spokesperson, you usually try to motivate people. And then if you see more and more cases where people get into trouble because of doing that, it makes yourself question your work a little bit more [. . .]. (Netherlands, I11)

Affective effects of criticism and harassment often include stress and frustration, sometimes sadness and disappointment, and in a few cases distress and anxiety:

It’s very difficult to read the comments and to just say, I don’t give a damn. No I must say, I will think about it all weekend, I was not crying, but I was like: “Should I answer, should I not answer, why are they touched, did I do right?” [. . .] It’s quite annoying or exhausting, so I’m happy not to face it every weekend. (Switzerland, I08)

Behavioral effects manifested in adapted communication strategies (e.g. avoiding social media platforms where backlash is more likely) and new work routines (e.g. group meetings to discuss difficult cases).

#### Effects on individuals, institutions, and society (system-level dimension)

Going beyond previous research, we also explored the system-level dimension of public backlash against science communication, differentiating between perceived effects on individuals, institutions, and society. Participants described a variety of person-level effects on others, including distress, anxiety, and loss of motivation. All participants reported cases or assumptions of self-censorship by individual scientists, though their accounts varied in form and severity: some described full withdrawals from contested research topics, others described scientists declining specific media requests or stepping back from particular platforms, and several described a more diffuse reduction in willingness to engage publicly:

The researchers were very nervous about talking about their research to the general public. Up to the point where they were not really interested in pursuing many communication options that would otherwise be available to them. One researcher I know who won’t engage in community events anymore. (Australia, I04)

Self-censorship was rarely described as total or permanent. Several participants noted that scientists who initially withdrew later returned to public engagement, and others observed a selection effect in which the most visible scientists are precisely those willing to absorb sustained hostility:

He actually announced that he will stop researching COVID. But, on the other hand [. . .] he has been really active, has continued to be really active on social media. Perhaps he held up for a couple of weeks, but he has continued with writing opinion articles and participating in events directed towards a general audience anyway. (Sweden, I13)

Several interviewees also described effects on the institutions they work for. However, most considered these effects less severe than those on individuals, since universities are more robust and better prepared for isolated expressions of criticism and harassment than individual scientists and communication professionals are:

The patterns are similar, but the good thing is that attacks [against the institution] are generally less emotional. That, in turn, is what makes individual attacks so hurtful. The people who do this have a terrible gift for phrasing things in such a way that they trigger feelings of concern and threat on an individual level. That cannot happen to the institution as a whole. (Austria, I02)

When asked about the impact of criticism and harassment on society, most participants were pessimistic. A common theme was a pronounced concern about growing societal polarization into pro- and anti-science groups. Some participants were also concerned that “the voice of science gets silenced” when “threats actually make the researchers stop doing what they do. That’s a democracy problem” (Sweden, I13).

#### Positive effects of public backlash (valence dimension)

The majority of backlash against communication professionals working at or with higher education and research institutions has negative consequences. However, all interviewees also mentioned positive effects. This illustrates the importance of considering a *valence* dimension of criticism and harassment (see Figure 2). Some participants noted, for example, that successfully handling backlash can feel rewarding:

Being a supporter can also be rewarding and an appreciated challenge: When you are a team and [. . .] have a good plan, then it’s thrilling to find the path to calm all people down and to say: “Okay, just let us have a reflection: That’s the answer and that will be the way to resolve the problem.” (Switzerland, I08)

While scientists were widely reported as withdrawing in the ways described above, we found little evidence of self-censorship among communication practitioners themselves. Many have become resilient and engage in counterspeech (see Nölleke et al., 2023). Nor do our analyses confirm concerns about severe reputational repercussions for universities, as few participants believed that harassment effectively damages the prestige of their institution:

Very often it’s still connected to a person and then our main focus is to support the person. So, I’m not worried about our reputation with a tweet like that. That’s not on top of my mind. (Netherlands, I11)

On the contrary, some participants noted that successfully dealing with public criticism is “actually beneficial to certain target groups in terms of reputation” (Germany, I01). Further positive effects include the development of training programs and increased alertness among university leaders:

In that sense it has had a good effect. Now at [UNIVERSITY], we put more focus on the preparing and protecting part instead of only the communication itself. It has been a positive effect that we’re spending more time on the ‘before’ part and also after you’ve communicated. (Netherlands, I11)

Some participants noted that not only have university leaders become more alert, but public awareness and media salience have also grown. This has prompted positive changes to their work ethos and routines:

The level of professionalism and seriousness has definitely increased. This has also put a little more pressure on us to improve constantly and keep on our toes so that we can continue to do a good job. (Germany, I09)

### Responses to backlash (RQ3)

Next, we explored how science communicators and institutions respond to public backlash. We focused on the “reactive” and “proactive” dimensions of responses (Veletsianos et al., 2018), following the stimulus–response rationale of research on how other epistemic authorities, such as journalists, deal with harassment and criticism (Holton et al., 2024).

#### Reactive and proactive coping strategies

Most approaches to coping with criticism and harassment against communication professionals, their institutions, and members of their institutions are *reactive*, responding to backlash only after it has occurred. For example, almost all participants said that they report or delete offensive social media posts that clearly violate netiquette. In severe cases, participants would also use more confrontational countermeasures, such as blocking users or taking legal steps. These reactions are typically firm—we found very few mentions of shying away, apologizing, or correcting their own statements. Only a minority of coping strategies are *proactive*, addressing harassment before it occurs. One such strategy is to deliberately avoid topics and platforms where greater incivility is to be expected.

Some responses are less one-directional than deleting posts and blocking users. For example, several participants said that they seek dialogue and exchange with critics:

For example, on social media, I would not block a response unless it is becoming notorious. In most cases, my social media [response] would be to keep on explaining to them so that they understand. (Kenya, I12)

However, participants also reported that they may not respond to criticism and harassment at all, instead staying “strategically silent.” The ability and strength to remain quiet appears to be one of the advantages of institutional over individual coping strategies:

We are much better to put backlash into perspective. That’s our core competence. We say that it will blow over. Scientists are not always able to do that, but some of them are. (Germany, I05)

#### Responses differ depending on legitimacy perceptions and sources of backlash

Across the range of reactive and proactive coping strategies, we found that the perceived legitimacy of backlash and the status of the source are particularly decisive for how participants and their institutions respond to criticism or harassment. The following quote from a participant in the United Kingdom captures this:

The first thing is to assess how high-profile is the person criticizing us. The second thing is to see if the critique is legitimate, if it has to do with a factual error that needs to be corrected. (United Kingdom, I14)

**Figure 3.**
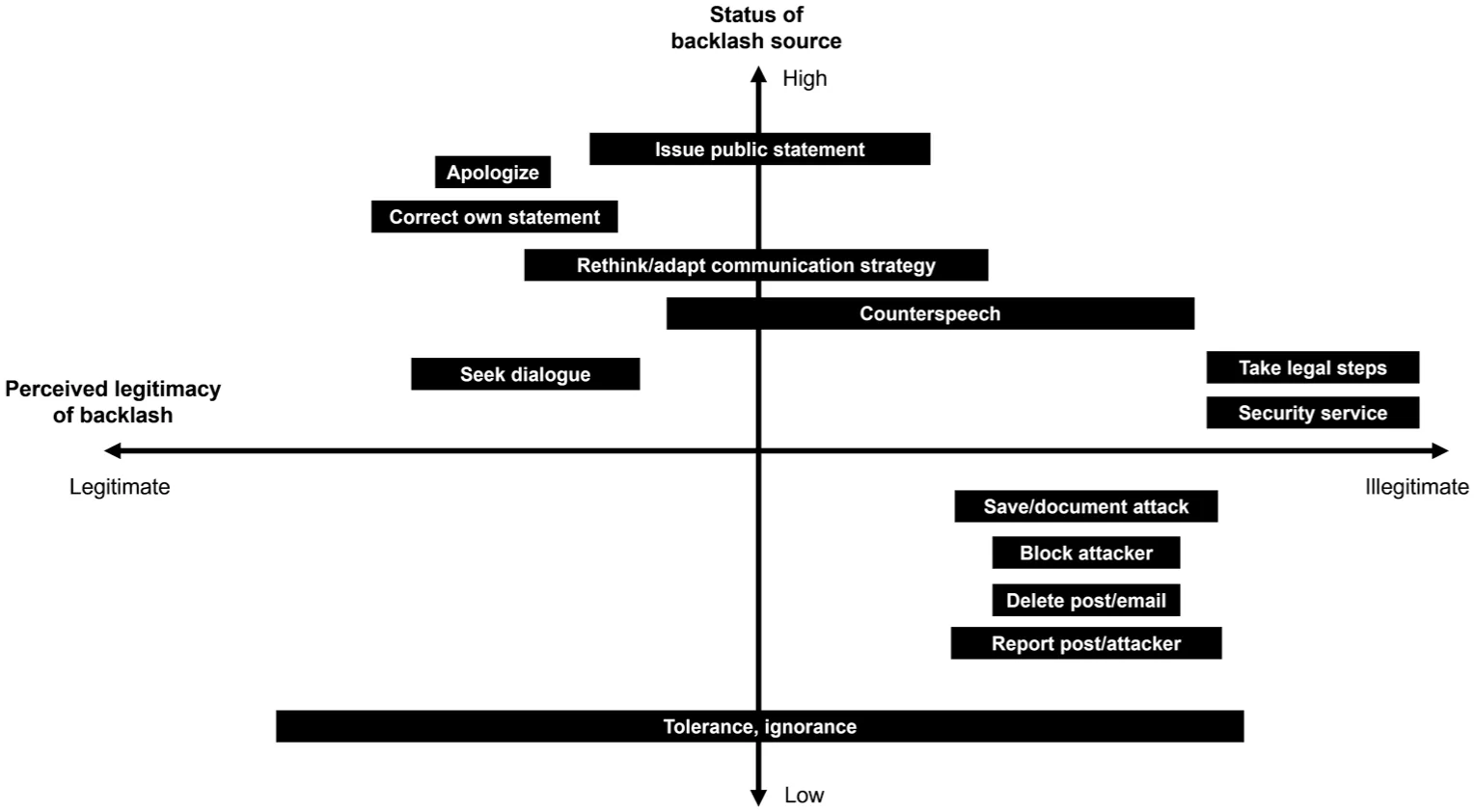
Responses to backlash depending on perceived legitimacy and source status.

We therefore mapped the different response strategies onto a two-dimensional space (see Figure 3). This analysis yields three main insights. First, backlash from high-status sources is more likely to elicit a response, as it has greater potential to cause harm. Several participants said, for example, that they would engage in counterspeech, issue public statements, and reconsider their communication strategy only in response to backlash from social media accounts with large follower numbers or from prominent institutions such as governments and media outlets:

If it’s [. . .] the kind of response that we got from the Filipino government, we may make a statement as we did. [. . .]. If it’s a low follower account, we don’t respond. If it’s a high follower account, we might respond. (United Kingdom, I14)

Second, the form of engagement with more influential sources depends on the perceived normativity of backlash. Non-normative, illegitimate harassment, for example, typically results in non-conversational strategies such as deleting posts, blocking attackers, or taking legal steps against perpetrators, whereas normative, legitimate criticism is more likely to motivate participants to seek dialogue and exchange:

It depends. If they just shout “Hey, you’re a left wing and I don’t like it,” that’s fine. If people have a concrete question where you can see that they actually want the question to be answered. So, if it’s an honest question and not an ironic question or a passive aggressive question, then we would usually try and answer it. And if it’s criticism, for example, ‘hey, your research integrity is at stake because of this’ [. . .], then we will definitely at least look into it and investigate internally. (Netherlands, I11)

Third, participants would generally not respond to backlash from low-status sources, regardless of whether it was normative or non-normative. Several strategic rationales lie behind this. One is that engaging with low-status critics and harassers may prompt others to chime in and amplify backlash. One participant described this in terms of “communication consequences assessment” (Germany, I05), referencing the concept of Technology Assessment (Grunwald, 2009). Another reason for staying silent in the face of harassment from less influential actors is that engaging is perceived as a fool’s errand:

We try not to engage too much because we are not necessarily going to change the person’s mind. They are often not reaching out to us for information. They are reaching out to us because they are angry or passionate about something. So, we do try not to encourage engagement because it actually does not help either party. (Australia, I04)

Staying silent in response to backlash from low-status sources—particularly when it involves non-normative attacks and threats—may be problematic. While reasonable from a strategic communication and crisis management standpoint, it risks establishing science–society dialogue as an exclusive conversation between influential actors and silencing public sentiments. We found few mentions of this dilemma; one exception was a participant who described ethical struggles over whether to engage with anti-science populists.

### Contextual factors of perceptions, effects, and responses (RQ4)

Our analyses show that the arguments, tone, volume, sources, and targets of backlash (RQ1), its attitudinal and system-level effects (RQ2), and responses to backlash (RQ3) are associated with several contextual factors. We distinguished five: (a) the route of backlash, (b) characteristics of the topic, (c) individual dispositions, (d) institutional conditions, and (e) the sociopolitical context.

#### Criticism and harassment vary across platforms

While a few participants reported cases of harassment at public events and on campus, the large majority of backlash against communication professionals occurs on digital platforms, particularly social media. This is consistent with several existing studies showing that these platforms have become the primary route of backlash against scientists and science communicators (e.g. Seeger et al., 2024). However, some participants identified differences between platforms. X (formerly Twitter) appears to be more conducive to backlash, partly because it harbors politicization of science issues, which in turn invite more harassment:

Twitter, it’s terrible. Twitter is the one media channel that politicians are using. And on the other hand, it’s a terrible medium. There’s a lot of hatred there. (Netherlands, I10)

Other platforms such as LinkedIn and Instagram are seen as less susceptible to non-normative backlash. This is consistent with surveys showing that these platforms rank among those where harassment of scientists is least common (Science Media Center Spain, 2024):

Compared to Twitter, Instagram is much more of a feel-good channel. I would rank them as follows: Twitter, Facebook, LinkedIn, a long pause, and then Instagram. (Austria, I02)

Yet almost all participants noted that much backlash—particularly intimidating and harmful backlash—happens “below the surface” via private communication, especially email: “You know I guess the most unhinged stuff is probably via e-mail” (Canada, I07).

#### Topic characteristics

Our analyses show that criticism and harassment are not per se limited to specific disciplines and research fields. Participants described backlash across numerous topical contexts. Environmental issues (particularly climate change) and health (particularly the COVID-19 pandemic) are the most prominent, but we also found mentions of gender studies, animal experiments, inclusive education, evolutionary theory, and farming and fishing.

#### Individual dispositions

Further contextual factors of backlash perceptions, effects, and responses include individual, institutional, and sociopolitical factors. Individual factors shaping perceptions of backlash include resilience, personal support structures, motivation to engage in outreach and withstand backlash, and conceptions of one’s role as a science communicator. Participants who see themselves as a public representative and “reputation guardian” (Campbell et al., 2006: 192) of their institution, for example, tend to be more resilient to backlash, viewing it as an occupational hazard.

#### Institutional context

Institutional factors shaping backlash perceptions, responses, and especially effects include the availability of counseling services and training programs that prepare scientists before they engage with the public, journalists, or politicians (Jupskås et al., 2025).

#### Sociopolitical conditions

Regional and national contexts also contextualize how communication professionals perceive and respond to backlash. A few participants reported threats from politicians in authoritarian countries:

Our research has been attacked this year by the Filipino government because they don’t agree with some of the facts that [a colleague from the communications team] stresses on their country profile, where she speaks about harassment and red tagging against journalists. (United Kingdom, I14)

However, we also found instances of political backlash in liberal democracies, such as the Netherlands, Switzerland, and Australia:

Some of our government representatives, like ministerial people, have made public announcements about the [INSTITUTION] taking money from the industry, which is very odd because they are meant to be our ministers. We are a governmental body, and when they attack us, it seems very odd because we are a part of them. (Australia, I04)

Further analyses highlight the importance of (perceived) polarization and local political opinion climates (see Hameleers and Van der Meer, 2025). The nitrogen crisis, for example, has made agricultural research a highly political topic in the Netherlands, while in Kenya, malaria research is a controversial issue:

In fact, the science communicators here face a number of hostilities, even when it has to do with issues about politics because you cannot separate science from the political arena. These things go together. (Kenya, I12)

This demonstrates that “the local” remains important in the contemporary global, networked science communication ecology. There is therefore no one-size-fits-all approach to dealing with backlash in science communication, “because nuances and perceptions are different in different countries” (United Kingdom, I14).

## 5. Discussion

We conducted one of the first studies on the increasingly relevant yet underexplored question of how communication professionals working at or with universities perceive and respond to public criticism and harassment. Our qualitative interview study with an international sample of 15 science communicators complements and extends a growing body of research on public backlash against science. We address key gaps in this literature by analyzing the role of university communicators as “boundary spanners” between sources and targets of backlash (Schwetje et al., 2020: 193), distinguishing between legitimate criticism and illegitimate harassment, and comparing multiple institutional and sociopolitical contexts. Our study also provides new insights into some of the most pressing dilemmas in current science communication research and practice: balancing the normative imperative for outreach against the risk of public harassment; mitigating illegitimate harassment without silencing potentially valuable public criticism; and finding the right balance between individual, institutional, and national response strategies (Nogrady, 2024).

### Key contributions

We highlight seven main results. First, we derived a new conceptual model of public criticism and harassment in science communication from our interview data (see Figure 2). It describes four central dimensions of public backlash against science communicators—types, effects, responses, and contextual factors—and relates them to one another. The model allows scholars and practitioners to disentangle the differential perceptions, effects, and responses to backlash and to situate their work within a broader set of analytical categories and processes.

Second, our analysis underscores the importance of conceptually distinguishing between the argumentative substance and the tone of backlash. This distinction is particularly important because our participants were often not fully aware of it: for most, tone and arguments were inextricably linked, and tone typically determined their legitimacy assessments. We conceive of this as an “unbalanced dialectic” and argue that it may undermine efforts to make university communication more receptive to legitimate public concerns, as uncivil but legitimate criticism may receive little attention while civil but illegitimate criticism may have disproportionate impact.

Third, we found that the “boundary spanning” function of university communicators (Schwetje et al., 2020: 193) has underacknowledged consequences for their workload and well-being. They must remain vigilant at multiple boundaries at once and, in crisis situations, respond to backlash from journalists, politicians, and the public simultaneously. Communication professionals and their institutions may also serve as scapegoats for alleged or actual failures of scientists and provide emotional or legal support to them. Our data suggest that this boundary work can be just as challenging as being personally targeted, as it may involve effects similar to those documented for attacks on scientists, such as distress, anxiety, or loss of motivation (Celuch et al., 2024; Väliverronen and Saikkonen, 2021a). Part of what makes this boundary work demanding is that backlash may target a colleague, the institution, or science itself, often without clear separation between these levels in any given incident—a layering that participants navigate in real time and that frequently shapes how they assess severity and craft a response.

Fourth, many participants showed notable resilience against harassment (see Nölleke et al., 2023). This cannot be explained solely with a “survivor bias” in our sample, which may have led to an underrepresentation of communicators with low resilience. We found ample mentions of positive effects—cognitive (heightened alertness), affective (rewarding experience of dealing successfully with backlash), and behavioral (optimizing response strategies). Some participants also reported positive organizational and societal effects, such as growing support from university leaders and increased public awareness of backlash against science. These findings suggest that science communicators, at least in the countries we studied, may not be as helpless or agency-deficient as public debate has often implied (see Veldkamp, 2021). That said, the volume and variety of negative effects remain substantial and warrant continued systematic research and countermeasures.

Fifth, we find preliminary evidence for a limited-effects model of public criticism and harassment at the institutional level. Many participants described detrimental effects on individual scientists and voiced concerns about the status of science in society, but doubted that backlash would have sustained impact on the reputation of their universities. This may reflect the sample’s concentration of institutions with established crisis communication functions, even where formalized backlash-handling procedures were absent.

Sixth, we show that two important determinants of responses to backlash are its perceived legitimacy and the status of its source (see Figure 2). Notably, we identified almost no response strategies for normative attacks (e.g. legitimate criticism about vested interests) from low-status sources (e.g. social media users). This may partly be a methodological artifact, as our interviews focused on more severe backlash and we recruited few social media managers. However, it may also point to limited receptiveness to concerns from the general public, albeit for plausible cost-benefit reasons.

Seventh, our findings suggest differences between countries. Communicators in “Global North” countries such as Sweden may face different challenges than communicators in “Global South” countries such as Kenya, including different controversial topics and different routes of backlash. Our data also indicate that the Netherlands and Germany may be better equipped against backlash, thanks to national contact points and many institutions—including, but not limited to, those of our interviewees—having standardized procedures and support structures. These cross-country differences underscore that harassment in science communication cannot be fully anticipated with one-size-fits-all solutions or centralized procedures, given the diversity of contexts and scenarios that require tailored responses.

These contributions should be read alongside several limitations. The relatively small sample of 15 communicators does not support statistical generalization, and country-level patterns should be treated as illustrative rather than representative. Our findings also rest on self-reported perceptions and capture how participants made sense of backlash rather than the motivations of those producing it. The cross-sectional interview design further means we cannot track how perceptions, effects, and responses evolve over time. A further caveat concerns our central concept itself: while we retain “backlash” as the most useful shorthand available, no single umbrella term precisely captures the breadth of phenomena it covers, from legitimate criticism to physical assault. Future conceptual work could sharpen this terminology, perhaps by developing a more differentiated vocabulary that distinguishes the various forms backlash can take.

### Future challenges

Our results also point to several directions for future research. Science communication professionals at universities play a significant role as boundary spanners between sources and targets of backlash and therefore deserve more attention in future studies. Such research could focus on sources with substantial political, financial, epistemic, or discursive power, since participants perceived backlash from these sources as particularly impactful. We accordingly recommend further scholarship and improved action on backlash from journalists, politicians, and (former) colleagues (see Egelhofer et al., 2026).

Future studies should also assess automated or coordinated harassment, as well as clustering dynamics and backlash accumulation around key events (e.g. publication of high-profile studies), topics (e.g. climate), and routes (e.g. platforms such as X). Such work would help anticipate times, issues, and platforms that attract more backlash—for example, through machine learning models—and could inform proactive training programs. These programs would help practitioners not only monitor issues where backlash is likely and prepare accordingly, but also actively seek out issues and platforms (e.g. LinkedIn or Instagram) with more constructive discourse dynamics.

## Supplemental Material

sj-docx-1-pus-10.1177_09636625261452891 – Supplemental material for Public backlash against science communicators: Conceptualization and qualitative analysis of perceptions, effects, responses, and contextual factors Supplemental material, sj-docx-1-pus-10.1177_09636625261452891 for Public backlash against science communicators: Conceptualization and qualitative analysis of perceptions, effects, responses, and contextual factors by Niels G. Mede and Sophia C. Volk in Public Understanding of Science

## Notes

1. These targets may overlap, yet they are conceptually distinct, as backlash against scientists or institutions does not necessarily entail rejection of scientific epistemology. We thus use the terms “science” and “anti-science” broadly, encompassing the organizations, individuals, and methods involved in the production and communication of scientific knowledge, as well as this knowledge itself.
