# Supplementary material for: Public backlash against science communicators: Conceptualization and qualitative analysis of perceptions, effects, responses, and contextual factors
Source: Public Underst Sci. 2026 Jun 3;35(7):866–90. doi: 10.1177/09636625261452891 (PMC13598313; doi:10.1177/09636625261452891)
Supplement: Supplementary file 1 [file sj-docx-1-pus-10.1177_09636625261452891.docx]

**Public Backlash Against Science Communicators: Conceptualization and Qualitative Analysis of Perceptions, Effects, Responses, and Contextual Factors**

Niels G. Mede^1*^ & Sophia C. Volk^2^

^1^Strategic Communication Group, Department of Social Sciences, Wageningen University & Research, Hollandseweg 1, 6706 KN Wageningen; Email: [niels.mede@wur.nl](mailto:niels.mede@wur.nl); Phone: +31 (0) 317 486 035; ORCID: 0000-0001-5707-7568

^2^Department of Media and Communication, Ludwig-Maximilians-Universität München, Munich, Germany; ORCID: 0000-0003-0887-8224

*Corresponding author

**Supplemental Material**

**Table of Contents**

[Appendix 1: List of Participants	2](#_Toc213491661)

[Appendix 2: Interview Guide	3](#_Toc213491662)

[Appendix 3: Codebook	7](#_Toc213491663)

[Appendix 4: Interview Excerpts Including Codings	13](#_Toc213491664)

**Appendix 1: List of Participants**

**Supplemental Table 1.** List of participants

| Interview number | Country | Sex | Interview length (min) |
| --- | --- | --- | --- |
| I01 | Germany | female | 45 |
| I02 | Austria | female | 55 |
| I03 | Switzerland | male | 55 |
| I04 | Australia | female | 60 |
| I05 | Germany | female | 60 |
| I06 | Switzerland | female | 60 |
| I07 | Canada | male | 60 |
| I08 | Switzerland | female | 70 |
| I09 | Germany | female | 60 |
| I10 | Netherlands | male | 60 |
| I11 | Netherlands | female | 55 |
| I12 | Kenya | male | 85 |
| I13 | Sweden | female | 60 |
| I14 | United Kingdom | male | 65 |
| I15 | Netherlands | female | 55 |

**Appendix 2: Interview Guide**

| **Dimension** | **Questions** |
| --- | --- |
| **Intro** | Thank you very much for participating in this interview, we appreciate it a lot. You are making an important contribution to our study on criticism, accusations and hostility in science communication. As we already explained in the email and the consent form, this interview is part of a study at [BLINDED].  Everything you say will of course be kept confidential and used only anonymously and for scientific purposes. If you feel uncomfortable talking about certain topics or experiences, feel free to say so - then we will simply skip the relevant questions.  I would like to record the interview so that we can analyze it more easily. Are you okay with that?  Two more notes: You can stop the interview at any time and also ask for the recording afterwards and the transcript to be deleted. We will be happy to send you the transcript as soon as possible; you can then make any changes you wish. We will not start the analysis until you have approved the transcript. Do you have any questions before we start? |
|  |  |
| **Work and role** | To begin with, I would be interested to know in general terms: What tasks do you carry out in your work?   - What does a typical workday or week look like, for example? - What media and channels do you use? |
|  | How do you see your role as a science communicator in society? To what extent do you have an important/special role? |
|  | To what extent do you perceive an obligation, expectation, or a certain pressure from the public in your role as science communicator? |
| **Vignette** | Science communication can use different channels and formats, and cause very different reactions, both positive and negative. Today, we are particularly interested in the latter. [SHOW VIGNETTE]  When I publish social media posts about recent research on the [UNIVERSITY] Twitter/X account, I sometimes get comments like: ‘Why does your press release say nothing about study limitation X? Why is your communication not transparent about the fact that your research is funded by Y?’    Any time language researchers from the [UNIVERSITY] publish a study on gender-inclusive language — anyone in linguistics can tell you the same story — they get endless hatred and sometimes even vague death threats. It’s anonymous people e-mailing them from weird accounts saying ‘I hope you die’.    Lately the online right-leaning blog [NAME] published a very aggressive commentary. It said something like: ‘The [UNIVERSITY] is littered with left winger hate groups that stop anyone from expressing an opposing viewpoint on their Twitter and LinkedIn channels.’  These were just a few examples; there are certainly many other types and forms of criticism and hostility. These can come from journalists, politicians, or citizens. Have you ever experienced or observed something like that? Can you describe it? |
| **Observed and experienced backlash** | Thinking back, what was the worst hostility you have experienced or observed? |
|  | Have you ever observed that **colleagues in your department and/or researchers at your university** were targets of hostility?   - If so, how did the hostility manifest itself? Can you describe your observations? How often does something like this happen? - Have you also observed threats of violence or even death? |
|  | Have you also experienced hostility against [**organization of interviewee**]?   - If so, how did the hostility manifest itself? Can you describe your observations? How often does something like this occur? |
|  | Have you personally (also) experienced hostility that was explicitly directed **against yourself**?   - If so, how did the hostility manifest itself? Can you describe your observations? How often does something like this happen? |
|  | Now I would like to talk about the content: Whether it's hostility **against yourself, colleagues** in the communications department, **researchers at your university**, or [**organization of interviewee**] in general: On which topics do you experience more hostility? Which ones less so? |
|  | If you now think of **less serious criticism**, like in the first example I showed earlier: Do you feel that it was partly justified? To what extent could it be that the targets of the criticism are partly to blame? |
| **Routes of backlash (arenas, platforms, etc.)** | Recall situations in which you have experienced hostility against **yourself, colleagues, researchers at your university**, or [**organization of interviewee**] once more: Where did these take place? Through which ways, channels, media? |
|  | Are there particular media or platforms where hostility is communicated more frequently?   - What role do digital media play? |
|  | Do you also observe or experience non-public hostility, e.g. in the form of letters, e-mails or private messages? |
|  | Are there certain actors who articulate hostility particularly often or particularly extensively?   - What do you think are their motives? - Have you ever had any personal contact with such actors? |
|  |  |
| **Effects** | Think about situations in which you have experienced hostility against **yourself, colleagues** in the communication team, **researchers at your university** or [**organization of interviewee**] once more: What effects and consequences can these have? |
|  | If **you yourself** have also been the target of hostility: What did this trigger in you personally?   - Mental distress? Stress? - Different/less communication? - Work motivation? - Resilience developed? Dullness? |
|  | If hostility targeted [**organization of interviewee**]: What was the impact?   - Loss of reputation? - Different communication strategy? |
| **Response** | Now think about hostility against **yourself** again: How do you deal with that?   - Do you talk about it with superiors, the university management, colleagues in your department or colleagues outside your university? Why (not)? - Does it help you to talk about it with friends, families, colleagues? Why (not)? - Have you ever used any support/counseling services? If yes: From whom? If no: Why not? |
|  | When [**organization of interviewee**] is exposed to hostility: How do you yourself or colleagues in the communications department deal with it?   - What are the best, most helpful, most effective, most proven solution strategies and measures? |
|  | Regardless of whether it is hostility against **yourself** or [**organization of interviewee**]: Do you or [**organization of interviewee**] actively respond to hostility? Why (yes/not)?   - Reply? Engage in counterspeech? - Legal action? - Block accounts? |
| **Institutional Support** | Are there any information or support services that you can access? If yes, which ones?   - Places or persons at [organization of interviewee], external contact point, advice, guide, etc.? - Have you ever used such offers? - Where/what is the first point of contact you can turn to when faced with hostility? - What is the role of university leadership, legal department? Other relevant actors? |
|  | What do you think of these offers? Helpful? Effective?   - If you have already used support services, which one was the most helpful? Which one was the least helpful? Why? |
|  | Do you wish there were (further) support services? If yes, which ones? From whom?   - How could existing offers be improved? |
| **Communicative Support** | Do you as a communicator or the communication department also receive requests for support in dealing with hostility from affected researchers or the university management?   - If so, has your department developed communication guidelines, training courses, advisory services, etc.? Are these perceived as helpful? Why (not)? - If no, why not? Do you think this will change in the future? - How do you perceive your role in such a situation? |
|  | If you now reflect again: Does your view of your work change as a result of observations or experiences of hostility? If so, to what extent?   - Does this do anything to how you perceive yourself as a science communicator? In what way? - Do you look differently at your role in society? In what way? - How does this change your role as a mediator/link between researchers and society? |
| **End** | That's it from my side now. Do you have any comments on the subject? Is there anything else you would like to say?  *If necessary, encouraging closing statement by interviewer in case the IV becomes too burdensome or ends depressingly, e.g.:*  *“We know from survey research that the vast majority of the public views science very positively and trusts it. So the voices that express hostility and criticism are in the minority – as incriminating as they can unquestionably be.”*  *“The fact that you face hostility like the ones we've talked about deserves respect and recognition; you're making an important contribution!”* |
|  | Thank you very much for the interesting conversation. Are you interested in a short summary of the results? Then we would be happy to send you one. |

**Appendix 3: Codebook**

**Target and source of hostility**

Source

Trolls

Journalists

Politicians

Society/members of the public

Other (e.g., scientists)

Target

Institutions

Rector/leadership

Comms colleagues

Scholars

Interviewee

Friends/family

Other (e.g., politicians, science journalists)

**Work and role understanding**

Role description

Crisis communicator/preventor

Watchdog/critical voice

Advocate

Service partner

Science translator/disseminator

Bridge/boundary spanner/mediator

Reputation guardian/ambassador

Enabler/empowerer

Job description

Media use in everyday work

Other (e.g., normative stance)

**Observed and experienced hostility**

Perception/assessment

Normative evaluation

Illegitimate

Legitimate

Crisis potential/severity (e.g., impact, influence of source)

Assumed reasoning/attitudes/motivations of source

Other

Topics

Covid

Environment and climate

Gender

Inclusivity

Energy

Public health

Conspiracy

Animal experiments

Social freezing/reproduction

Genetic engineering

Race

Other

Occurrence

Unclear/vague/varies

Frequent

Sometimes

Very rare

Has not happened

Type of Hostility

Non-specified attack

Aggression

Insult

Threat

Chicanery (e.g., trolling)

Discrimination (e.g., racism)

Defamation (e.g., slander)

Physical attack/violence

Other

**Routes of backlash (arenas, platforms, etc.)**

Non-mediated

Face-to-Face (e.g., events)

Telephone

Letters

Other

Mediated

Press release

Website of Institution

Radio

TV

Newspaper/magazine

Comment sections on news portals

Social media

Twitter

Facebook

LinkedIn

Instagram

YouTube

TikTok

Social Media (unspecified)

Other

Blog

Messaging service

E-mail

Newsletter

Other

**Individual-level effect**

Affect

Entertainment

Motivation/commitment (e.g., to communicate publicly)

Stress

Concern

Fear

Frustration

Other

Cognition

Awareness

Resilience

Change in self-image/role

Knowledge/competence

Other

Behavior

Self-de-platforming

Agency/self-efficacy

Self-censorship/silencing

Other

No Effects

Other

**Organizational-level effects**

Silencing/hesitation (e.g., avoiding specific topics) [organizational-level]

Organizational de-platforming

Concern of lawsuits against university

Impact on reputation

Sensitization of uni leadership

Other

**System-level effects**

Silencing or hesitation (e.g., avoiding specific topics) [system-level]

Research activities

Discourse quality (e.g., polarization)

Other

**Response strategies**

Other actions related to attack

Other

Taking legal steps

Save (e.g., screenshot)

Avoid/refute attack

Reporting/blocking attacker/post

Deleting post/comment

Refuting attack/counterspeech

Keeping quiet/doing nothing/strategic silence

Avoiding/disconnecting (e.g., rejecting/ignoring)

Address attack

Answer question/searching dialog with attacker/moderating

Accusing attacker

Ridiculing attacker (e.g., using sarcasm, irony)

Correcting own statements

Apologizing

**Support measures**

Descriptions about support measures

Desired support measures

Limits of support (e.g., loyalty conflicts, lack of resources, no demand, no backup)

Institutionalization of support (e.g. emergency contact, standardized processes)

Evaluation of support

No support measures

Individual-level support mechanisms

Humor

Resilience

Social coping (e.g., talking with peers)

Other

Institutional-level support mechanisms

Preventive

Courses/workshops

Monitoring

Guidelines

Enabling/advising on communication

Peer exchange (inc. external networks)

Regular contact/gatherings

IT security

In situ

Peer exchange (incl. external peers)

Communication advice or support

University and/or leadership backing

Legal counsel

Listening/informal conversations

(Psychological) counsel

Filtering and analysis

A posterior

Supporters

Interviewee or comms department

University leaders

Legal department

National contact point (e.g., SciComm Support)

Supervisors

Police

Friends/Family

Colleagues

Other

Counselor/psychologists/therapists

Security

University/institution

IT department

**Contextual background**

Other

Perception of public opinion on science communication (e.g., trust in science, polarizations)

Comparison or reference to other contexts/countries/institutions/times

**Appendix 4: Interview Excerpts Including Codings**

**Supplemental Figure 1.** Interview excerpt with codings focusing on backlash perceptions

**Supplemental Figure 2.** Interview excerpt with codings focusing on backlash effects

**Supplemental Figure 3.** Interview excerpt with codings focusing on response strategies
